# Nontriplet feature of genetic code in *Euplotes* ciliates is a result of neutral evolution

**DOI:** 10.1073/pnas.2221683120

**Published:** 2023-05-22

**Authors:** Sofya A. Gaydukova, Mikhail A. Moldovan, Adriana Vallesi, Stephen M. Heaphy, John F. Atkins, Mikhail S. Gelfand, Pavel V. Baranov

**Affiliations:** ^a^Faculty of Bioengineering and Bioinformatics, Lomonosov Moscow State University, Moscow 199911, Russia; ^b^A. A. Kharkevich Institute for Information Transmission Problems RAS, Moscow 127051, Russia; ^c^Laboratory of Eukaryotic Microbiology and Animal Biology, School of Biosciences and Veterinary Medicine, University of Camerino, Camerino 62032, Italy; ^d^School of Biochemistry and Cell Biology, University College Cork, Cork T12 XF62, Ireland; ^e^Department of Human Genetics, University of Utah, Salt Lake City, UT 84112

**Keywords:** ciliates, genetic code, ribosomal frameshifting

## Abstract

The triplet nature of the genetic code is considered a universal feature of known organisms. However, frequent stop codons at internal mRNA positions in *Euplotes* ciliates ultimately specify ribosomal frameshifting by one or two nucleotides depending on the context, thus posing a nontriplet feature of the genetic code of these organisms. Here, we sequenced transcriptomes of eight *Euplotes* species and assessed evolutionary patterns arising at frameshift sites. We show that frameshift sites are currently accumulating more rapidly by genetic drift than they are removed by weak selection. The time needed to reach the mutational equilibrium is several times longer than the age of *Euplotes* and is expected to occur after a several-fold increase in the frequency of frameshift sites. This suggests that *Euplotes* are at an early stage of the spread of frameshifting in expression of their genome. In addition, we find the net fitness burden of frameshift sites to be noncritical for the survival of *Euplotes*. Our results suggest that fundamental genome-wide changes such as a violation of the triplet character of genetic code can be introduced and maintained solely by neutral evolution.

## Significance

In this work, we provide compelling evidence that *Euplotes* genetic code violates the triplet nature of the genetic decoding that was thought to be universal. Thus, *Euplotes* possess the most extreme example of genetic code variation described so far. The nontriplecy arises from abundant ribosomal frameshift sites with no regulatory function, where stop-codons distant from the 3′ transcript end specify +1 or +2 ribosomal frameshifting with high accuracy. We show that this violation of the triplet coding in *Euplotes* is brought about and further maintained by neutral evolution rather than selective processes but still is irreversible.

The sequential nonoverlapping triplet nature of genetic decoding was established by Crick, Brenner and their colleagues in early 60s (1). Almost all proteins are encoded by such sequential nucleotide triplets, codons. Thus, the decoding ribosome moves along mRNA in one of the three-periodic phases known as the reading frames. Errors in maintaining the reading frame are more detrimental than missense errors as they affect the entire downstream part of the protein (2). Consequently, spontaneous shifts between reading frames are highly infrequent (3). As the accuracy of triplet decoding is sequence-dependent, frameshifting-prone sequences are selected against in protein-coding genes (4, 5). However, the sequence dependence of frameshifting efficiency enabled evolution of genes that exploit this phenomenon to regulate their expression in the process known as programmed ribosomal frameshifting (6). Although genes requiring ribosomal frameshifting for their expression have been found in most organisms, such genes are generally extremely rare, though common in viruses (7) and transposable elements (8). To achieve higher efficiency, programmed ribosomal frameshifting often requires the presence of elaborate stimulatory signals such as RNA pseudoknots altering progression of the ribosome (9, 10) or nascent peptides interfering with the ribosome function from within (11, 12). Even with the assistance of such stimulators, the efficiency of ribosomal frameshifting is usually lower than that of the competing triplet decoding (6). Thus, the product of ribosomal frameshifting is synthesized in addition to the product of standard translation.

**Fig. 1.**
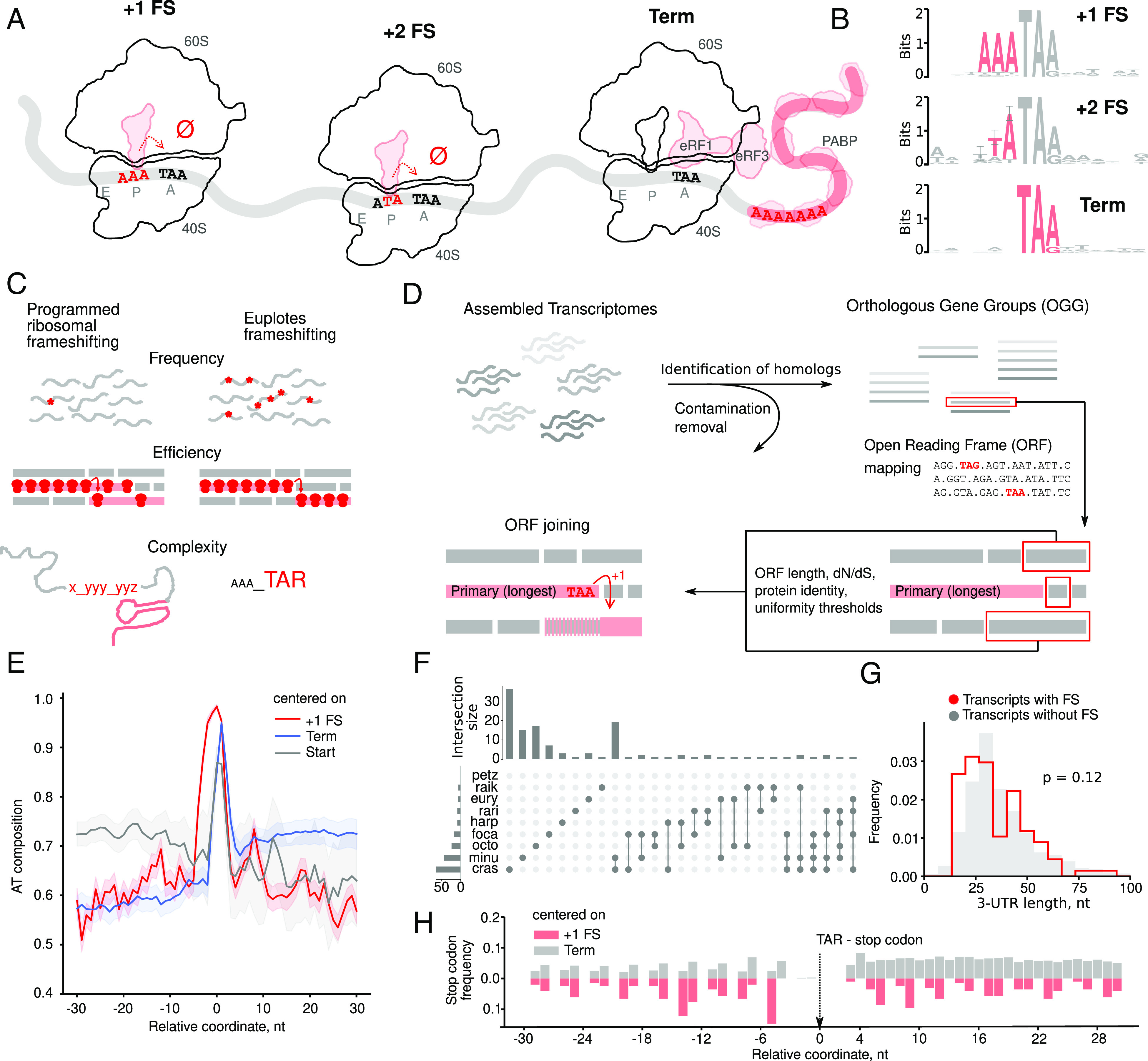
*Euplotes* transcriptomes and identification of frameshift sites (T specifies uridine in RNA). (*A*) The proposed mechanism of ribosomal frameshifting for codons supporting tRNA repairing with either +1 (A*AA T*AA) or +2 (AT*A TA*A) codons (in italics) and translation termination in *Euplotes* spp. Conserved bases are in red. (*B*) Sequence LOGOs of frameshift sites and terminating stop codons contexts. (*C*) Properties of ribosomal frameshifting in *Euplotes* spp. in contrast to programmed ribosomal frameshifting. (*D*) Schematic representation of the algorithm for identification of frameshift sites. (*E*) Positional AT-content relative to start and stop codons (FS and terminators) centered at zero. (*F*) UpSetR representation of the distribution of identified frameshift sites across species. Rows correspond to the species and columns to the number of unique or shared frameshift sites. (*G*) Distributions of 3′ UTR lengths (nt) in transcripts with (red) and without (gray) frameshifts. (*H*) Stop-codon frequency around frameshifting (red) and terminating (gray) stop-codons.

Ribosomal frameshifting observed during mRNA translation in ciliates of the genus *Euplotes* (13–17) is often described as programmed ribosomal frameshifting, but, as we will argue below, strikingly contrasts to the programmed ribosomal frameshifting in other organisms. It has been proposed to occur whenever a stop codon is encountered by the ribosome (Fig. 1*A*). Unlike programmed ribosomal frameshifting, it is highly efficient with virtually no products of termination at stop codons at internal positions being detected (13). Termination of translation occurs only near the 3′ ends of mRNAs in close proximity to the polyA tails (13) similarly to the situation in those species where all three stop codons have been reassigned to code for amino acids (18–23) as outlined in ref. 24. Therefore, ribosomal frameshifting has been suggested to be a part of the standard *Euplotes* genetic code (13) (Fig. 1*C*).

It is not clear, however, how such a nontriplet feature of the *Euplotes* genetic code has emerged and which processes enable its persistence. To address these intriguing questions, we explored the evolution of frameshift site (FS) occurrences across genomes of several *Euplotes* species.

## Global and Local Alterations of the Genetic Code

The standard readout of the genetic information could be altered globally, affecting genetic code of an entire organism, or locally, affecting decoding of a specific mRNA (25). Global alterations involve a change of a molecular component that is required for decoding of most or all mRNAs in the cell or an organelle. For example, the loss of a gene encoding release factor 2 in vertebrate mitochondria resulted in the reassignment of UGA stop codon to tryptophan, so that all UGA codons in coding regions of mitochondrial mRNAs are decoded as tryptophan (26). While most codon reassignments involve stop codons (20, 27), sense codon reassignments have been also observed (28).

In addition, the meaning of a codon could also be altered locally in a specific mRNA. For example, the presence of a special RNA secondary structure, SECIS element, in the 3′ UTR of a eukaryotic mRNA can redefine a specific UGA codon in that mRNA to encode a selenocysteine (29). Selenocysteine is incorporated into the proteins synthesized from only 25 human genes whose mRNAs contain SECIS elements (30); mRNAs from other human genes lack SECIS and do not encode selenoproteins. An extreme example is the selenoprotein P mRNA in bivalve molluscs where 132 UGA codons are decoded as selenocysteines (31). To distinguish between global and local alterations of codon meanings, a change in codon meaning that affects the entire genetic code is termed *codon reassignment*, while a site- or mRNA- specific change of meaning is termed *codon redefinition* (25, 32). Dynamic codon redefinition is an instance of a more general phenomenon called *recoding* which encompasses numerous translational deviations from the standard genetic code occurring in the decoding of specific mRNAs with no overall effect on the genetic code (25, 33–35).

## Programmed Ribosomal Frameshifting

One of the recoding mechanisms is programmed ribosomal frameshifting which is extremely rare in cellular genes (6). For example, in humans, translation of only a handful of genes of nonviral origin is known to utilize ribosomal frameshifting. These are three paralogous genes encoding ornithine decarboxylase antizyme whose regulation via polyamine-dependent +1 ribosomal frameshifting is nearly universal (36). +1 and –2 ribosomal frameshifting is also suspected to occur in the expression of two paralogous genes *ASXL1* and *ASXL2* (37). The three genes that use –1 frameshifting are of the viral ancestry, i.e. *PEG10*/*EDR* (38, 39), *PNMA3*, and *PNMA5* (40). While there were reports of –1 frameshifting in human genes of nonviral origin (*CCR5* (41) and *ATP7B* (42)), they appeared to be due to misinterpretations of artifacts of the reporters used (43–45). The best studied bacterium, *Escherichia coli*, has only three genes of nonviral origin whose expression is currently known to utilize frameshifting, *prfB* (46–48), *dnaX* (49–51), and a more recently discovered *copA* (42). Even if these genes are combined with sequences of prophages and Insertion Sequence elements, the programmed ribosomal frameshifting is estimated to occur on average in only four genes per bacterial genome (52). Ribosomal frameshifting is more common in viruses, for which it provides an attractive mechanism to produce more than one product from the same mRNA, to maintain a fixed stoichiometric ratio between its products or for regulatory purposes (53), and to yield a more compact organization of protein-coding information (7). However, even in viruses, frameshifting rarely occurs at more than a single location per genome.

The efficient frameshifting may also be observed in so-called pseudo-pseudogenes (54, 55), genes that are expressed despite having nonsense or frameshift mutations. The latter happens because the sequence downstream of a frameshift-causing indel mutation is translated in a new frame and has not been optimized by evolution for accurate triplet decoding, thus containing frameshifting-prone sequences (4, 5).

## Abundant Ribosomal Frameshifting in *Euplotes* spp. Is Not Programmed Ribosomal Frameshifting

The frequency of ribosomal frameshifting in *Euplotes* spp. is in striking contrast with the frequency of programmed frameshifting. This was initially observed by Lawrence Klobutcher and Phil Farabaugh (15) who noted that, at the time when sequences of only 67 genes from *Euplotes* species were available, frameshifting was reported in four genes (56–59) suggesting that it occurred in more than 5% of *Euplotes* genes. Subsequent ribosome profiling and proteomics studies did confirm the high frequency of ribosomal frameshfting in *Euplotes* spp. by identifying thousands of instances of frameshifting and revealing that about one-fifth of all *Euplotes* genes use frameshifting in their expression, with some genes having up to eight frameshift sites (13, 16, 17).

In addition to its high frequency, frameshifting in *Euplotes* spp. is highly efficient and provides deterministic readout, again arguing that it is a feature of a *Euplotes* standard genetic code. Indeed, all known cases of programmed ribosomal frameshifting are not 100% efficient (6). Only a fraction of ribosomes shift the reading frame, while the remaining and usually a larger proportion, continue translation in the same reading frame or terminate when a stop codon is a part of the frameshift site. This optionality is at the core of the functional role of programmed frameshifting in gene expression, as it creates a bifurcation in the process of mRNA decoding yielding two different products of the same gene (34). Even when only one of these products is a functional product, the sensitivity of the frameshifting efficiency to the cellular environment provides an opportunity for regulation. The best-known eukaryotic example is the polyamine-sensitive +1 frameshifting required for synthesis of the protein antizyme. This is a key part of a negative feedback loop, since antizyme is a negative regulator of polyamine synthesis and transport (10). A similar negative control loop operates in the *prfB* gene encoding bacterial release factor 2 (RF2) (46). In decoding RF2 mRNA, frameshifting competes with termination at a UGA stop codon which is recognized exclusively by RF2. Thus, a drop in the RF2 levels leads to increased frameshifting efficiency which in turn is required for RF2 synthesis.

The situation with ribosomal frameshifting in *Euplotes* spp. is clearly different. A ribosome profiling study did not reveal any significant drop in ribosome densities downstream of frameshift sites (13), that would be expected if the frameshifting efficiency was substantially different from 100%. Thus, unlike programmed ribosomal frameshifting, the frameshifting in *Euplotes* spp. is deterministic, as it does not compete with triplet decoding. Hence, a failure of the ribosome to shift could be considered as an error in the translation process, exactly as spontaneous frameshifting during triplet mRNA decoding in other species.

Furthermore, programmed ribosomal frameshifting requires the presence of stimulatory signals increasing its efficiency, such as RNA secondary structures (9, 60, 61), complementary interactions between ribosomal RNA and mRNA (62, 63) or nascent peptides (12, 64), or protein factors interacting with mRNA (65–67). Sequences known to trigger highly efficient ribosomal frameshifting in the absence of additional stimulators are known only in yeast (68). This largely is enabled by a severe imbalance in the concentration of in-frame and out-of-frame tRNAs, as in the frameshift site in the TY1 element consisting of CUU_AGG_C. Only one gene copy of AGG-decoding tRNA exists in *Saccharomyces cerevisiae* while there are sixteen copies of genes for tRNA recognizing the +1 GGC codon (69). This imbalance favors decoding of the +1 frame codon (69). Sequence requirements for frameshifting in *Euplotes* spp. are even weaker. Seemingly, all that is needed for frameshifting is either of the two stop codons, UAA or UAG [with UGA reassigned to cysteine (70)]. In early reports, all identified *Euplotes* frameshifting sites had the AAA codon preceding the stop, which prompted Klobutcher and Farabaugh to suggest that Lys-tRNA decoding the AAA codon had some special properties enabling ribosomal frameshifting at this codon via repairing with overlapping +1 AAU codon (15). However, subsequent high-throughput studies have revealed that while the AAA codon is by far the most frequently used codon in the frameshifting sites, it is not absolutely required for frameshifting. However, the identity of the codon preceding stop codons determines the mechanism of frameshifting, with AUA_UAR resulting in a +2 frameshifting, presumably via repairing of tRNA decoding AUA with the identical overlapping codon in the +2 frame (13). No additional signals or stimulators were found to be required for frameshifting, again suggesting that frameshifting is a standard default meaning of stop codons in the *Euplotes* genetic code (13).

This raises an immediate question. If the standard meaning of stop codons in the *Euplotes* genetic code is frameshifting, how does it terminate protein synthesis? Here, the situation in *Euplotes* spp. seems to be similar to that occurring in species with recently discovered genetic codes where all stop codons are reassigned to code for amino acids (18–23). It has been shown that in ciliate *Condylostoma magnum* the same stop codons are used as terminators, but in a strictly position-specific manner (18, 20). Ribosomes terminate only in close proximity to polyA tails, with 3′ UTRs in *C. magnum* being very short. Changes in the sequence of a specific tRNA and release factor have been recently attributed to the reassigned stop codons in trypanosmatid *Blastocrithidia nonstop* which includes position-specific meaning of UAA codon (22). The situation is similar in *Euplotes* spp. with only a single exception found so far, where termination takes place far upstream of the polyA tail (13). This exception is a mRNA encoding a selenoprotein. As mentioned earlier, selenocysteine incorporation requires the presence of a SECIS structure and thus 3′ UTR is sufficiently long to accommodate this structure. Possibly, in this case the polyA proximity to the terminating stop is steric rather than purely sequence-length dependent and it is likely that the formation of SECIS structure brings the two into proximity. The requirement for polyA proximity is most likely due to involvement of polyA binding proteins (PABPs) in the process of termination. The stimulatory effect of PABPs on termination is well documented across a variety of biological systems (71–74), and ciliates likely represent an extreme case of such a requirement. Indeed the ~100% efficiency of frameshifting in *Euplotes* suggests that it does not compete with termination as happens in most other cases involving frameshifting at stop codons (75). This is also indirectly supported by experiments that tested the stop-codon specificity of *Euplotes* eRF1 in yeast (76). A hybrid release factor needed to be created where the *Euplotes* eRF3 recognition domain had been replaced with the yeast version. Presumably this alleviated the requirement for the polyA proximity.

More generally, ciliates are famous for extravagant ways to organize and express their genetic material, such as multiple nuclei and extensive structural genome rearrangements during transfer of genetic information between nuclei such as gene unscrambling (77–79), exceptionally extra short introns (80–82), as well as exceptionally frequent stop-codon reassignment (25, 27). Likely, the strict positional dependence of translation termination is one of the features enabling this reassignment, as stop codons in the middle of mRNAs would be expected to be highly inefficient and would need to be resolved either via codon reassignment or ribosomal frameshifting as in *Euplotes* spp. (83). While position-specific termination and frequent genetic code alterations in the forms of codon reassignment and standard frameshifting, seem to be connected, the direction of their causality remains to be elucidated.

## Results

### Detection of Frameshift Sites.

We have sequenced transcriptomes of nine species from six major clades of the *Euplotes* phylogenetic tree (*SI Appendix*, Fig. S3): freshwater *Euplotes octocarinatus,* brackish waters *Euplotes harpa,* and marine *Euplotes focardii*, *Euplotes petzi*, *Euplotes euryhalinus*, *Euplotes rariseta*, *Euplotes minuta*, *Euplotes raikovi,* and *Euplotes crassus*. The transcriptomes were assembled and combined into 1,614 orthologous groups containing 4,903 sequences (*SI Appendix*, Table S1 and *Materials and Methods*). In all subsequent analyses, we considered only transcripts with identifiable orthologs, as we would not be able to assess the evolutionary trajectories of FSs from individual sequences. This also reduced contamination with sequences derived from other organisms found in the environment (*Materials and Methods*).

To detect FSs in these transcriptomes, we developed a systematic and unbiased procedure (*SI Appendix*, *Supplementary Methods*). We assumed absolute unambiguity of frameshifting in *Euplotes* spp., which means that ORFs following a frameshifting event were expected to be translated in a single reading frame. The procedure starts with the longest ORF that is then extended with adjacent or overlapping ORFs joined with either +1 or +2 frameshifting or stop codon readthrough (Fig. 1*D*). Candidate ORFs were detected using stringent criteria relying on minimal ORF length, high sequence similarity with their orthologs, signatures of purifying selection typical for the evolution of protein-coding sequences, and uniformity of the two latter characteristics along the candidate protein-coding sequence (*SI Appendix*, *Supplementary Methods*). To avoid arbitrariness in thresholds underlying these criteria, we considered these thresholds as parameters and fine-tuned them to obtain robust results. Subsequent validation using ribosome profiling data generated in *E. crassus* (13) demonstrated high specificity of the algorithm, with true- and false-positive discovery rates of 54% and 0%, respectively (*SI Appendix*, *Supplementary Methods*). Using this approach, we identified translated regions in the generated transcriptomes and found 3.9% of transcripts to contain FSs (8.3% orthogroups), i.e. 197 instances of +1 and 16 instances of +2 frameshift sites distributed across 192 sequences from the total 4,903 (Fig. 1 *B* and *F*). No stop codon readthrough events could be identified. No frameshifting events were identified in *E. petzi,* though manual analysis of alignments allows one to identify several frameshifts. This is likely due to stringent pipeline parameters combined with a small number of *E. petzi* transcripts with identifiable orthologs, as compared with other species. The set of predicted FSs and the predicted terminating stop codons (terminators) confirm previously reported features of *Euplotes* coding sequences, such as the prevalence of +1 shifts (predominantly at AAA preceding stop codons), short 3′ UTRs, enhanced AT-content in 3′ and 5′ UTRs and high frequency of stop codons in 3′ UTRs (Fig. 1 *E*, *G*, and *H*). The efficiency of termination is often influenced by the local context (84), however, we do not observe significant biases at FSs relative to terminating stops (Fig. 1*B*).

### Evolution of Frameshifts in *Euplotes* spp.

The unusually large number of tolerated, highly efficient FSs in *Euplotes* spp., a feature not observed in any other group of cellular organisms studied so far, calls for an evolutionary explanation. Although the current amount of data renders any population-based methods inefficient in this case, we can assess some basic evolutionary features of FSs, such as their general effects on fitness, from the FS gain and loss rates along the phylogeny.

**Fig. 2.**
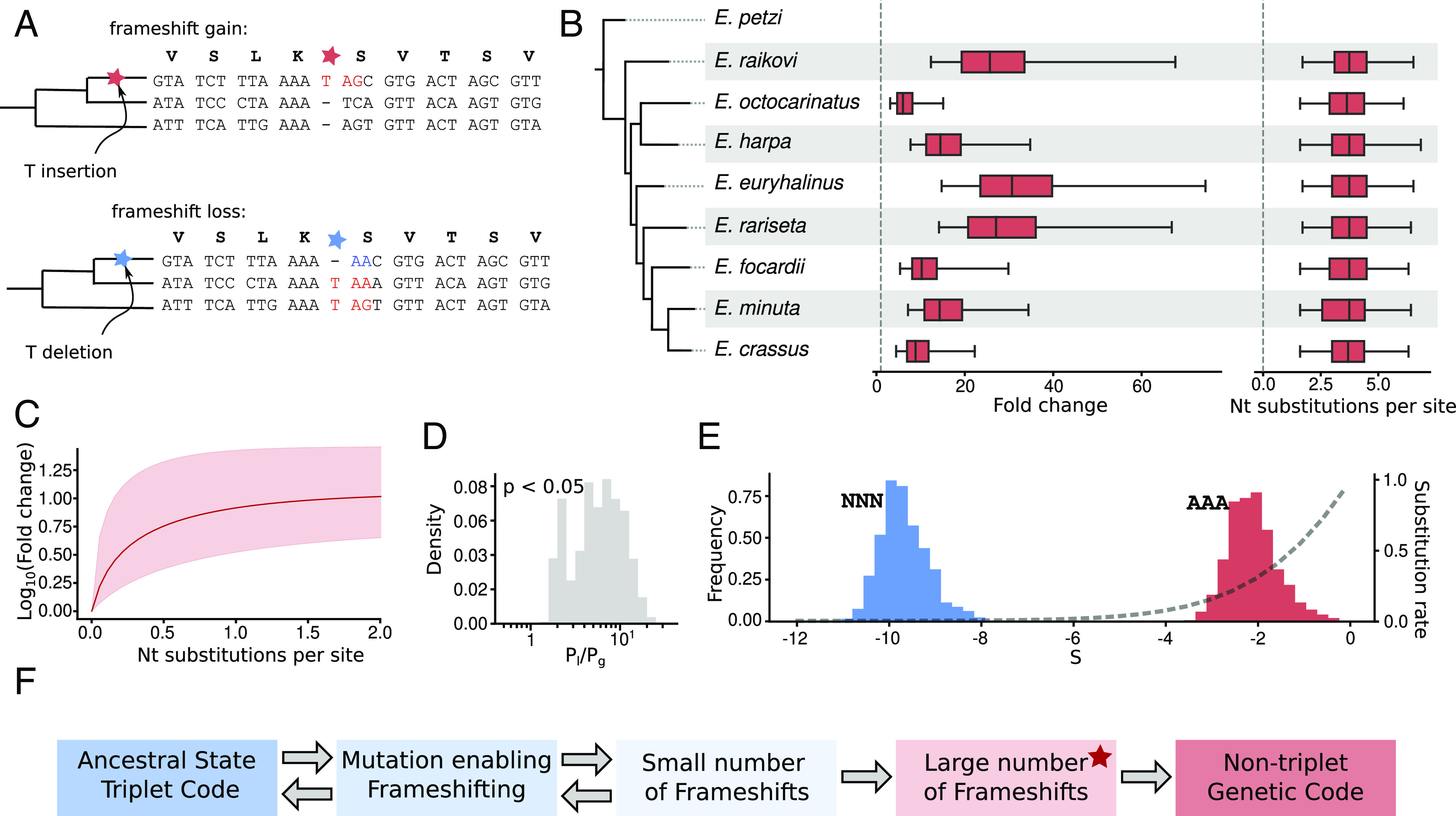
Increasing numbers of frameshift sites during the evolution of *Euplotes* spp. (*A*) Inference of gains (*Top*) and losses (*Bottom*) of FSs based on the ancestral states. (*B*, *Left*) Phylogenetic tree of studied *Euplotes* species. (*Middle*) Distributions of the fold changes in FSs numbers upon reaching equilibrium. (*Right*) Distributions of time intervals (in nucleotide substitutions) required to reach 95% of the FSs number expected at equilibrium. (*C*) Projected fold change of the number of FSs over time. Red shading indicates the 95% CI obtained from permutations. (*D*) Ratio of FS gain and loss probabilities for AAA_[T]AR contexts. (*E*) Permutation distributions of *S* values for selection against FSs arising in AAA_[T]AR contexts (red) and of upper bounds for *S* in non-AAA_[T]AR contexts (blue). Dashed line is the theoretical dependence of the normalized substitution rate for variants affected by selection and genetic drift on the *S* value. (*F*) Proposed scheme of the emergence and subsequent entrenchment of the nontriplet genetic code. A star indicates the current stage of the genetic code of *Euplotes*.

We inferred frameshift site gain and loss events based on the reconstructed ancestry (Fig. 2*A*). We found that the frequency of gains exceeds that of losses about ten-fold (39 vs. 4). This sharp asymmetry may be explained by positive selection, however, to test for selection we have to consider probabilities of FS gains and losses rather than the numbers of respective events. These probabilities depend on the number of contexts suitable for FS gains and the number of existing FSs (that may be lost). The evolution of FSs is likely to be context-dependent since ~72% of all gain and loss events occurred due to the insertion or deletion of T at AAA_[T]AR (underscore separates codons, R = A or G). Thus, we initially focused on the analysis of evolution of FSs conforming to this specific pattern. We define the probabilities of frameshift gains and losses as *P_g_* = *n_i_/K* and *P_l_* = *n_d_/F*, respectively, where *n_i_* is the number of observed insertions of T yielding new FSs, *K* is the number of ancestral AAA_AR sequence motifs, *n_d_* is the number of observed deletions of T leading to the loss of FSs, and *F* is the number of AAA_TAR FSs. As opposed to simple counts of FS gains and losses, FS loss probability exceeds FS gain probability by 3 to 30-fold (Fig. 2*D*, *P* = 0.028, permutation test). This discrepancy may be explained by selection against novel FSs combined with increased mutational pressure favoring FS gains. Concerning the latter, we observe the numbers of FS gain contexts to be on average 74-fold higher than the numbers of existing FSs. This inflates the probability of FS-gain mutations compared to FS losses (85). The selection against FSs may be estimated from differences in the observed probabilities of FS gain and loss (*Materials and Methods*), with the scaled selection coefficient calculated as the average *S = 4sN_e_* of FSs, where *s* is the selection coefficient and *N_e_*, the effective population size (85).

The calculated *S* (Fig. 2*E*) differed for frameshifts in the AAA_[T]AR and non-AAA_[T]AR contexts, and while *S* for frameshifts in the latter context are in the highly deleterious range (upper bound *S* = −10±1, the CI derived from a permutation analysis), frameshifts in the AAA_[T]AR context seem to be only slightly deleterious (S = −2 ± 1), which is consistent with fitness-reducing FS effect in *Euplotes* spp. that may be due to ribosome pausing (13). Indeed, the observed *S* = −2 may result in both negative selection and drift influencing accumulation of mutations, whereas *S* = −10 effectively indicates only negative selection acting against FSs arising in the NNN_[T]AR contexts (Fig. 2*E*) (85).

Thus, the large number of contexts suitable for FS gains compared to the numbers of already existing FSs yields a relatively large mutational target size for FS accumulation, which counters selection against FSs in favorable contexts. But are these processes at equilibrium and, consequently, are the frequencies of euplotid FSs constant in time? And, if there is no equilibrium, how distant from it are *Euplotes* spp.? The following differential equation describes the change of the number of FSs over time where *u_g_* and *u_l_* are, respectively, the rates of gain and loss derived from their probabilities (*Materials and Methods*), and *K* and *F* are, as earlier, the (constant) number of ancestral AAA_AR sequence motifs and the (variable) number of current AAA_TAR FSs, respectively:

$$dF\;=\;(K\;-\;F)\;u_{g}dt\;-\;Fu_{l}dt.$$

Solving this equation (*Materials and Methods*) we projected changes in the number of FSs over time. At infinite time, this function reaches the upper asymptote which corresponds to the number of FSs when gains and losses are at equilibrium. We find the asymptote to be (depending on species) 5 to 25-fold larger than the current numbers of FSs (*P* < 0.0001, permutation test, *Materials and Methods*) (Fig. 2 *B*, *Middle*).

Next, we estimated the time required to reach equilibrium. As formally this time is infinite, we consider the effective equilibrium as the time point when the number of FSs is 95% of its asymptotic value. We found these times to be consistent across species and constitute 1.7 to 6.3 nucleotide substitutions per site on average (Fig. 2*B*), which is about 0.68 to 2.52 of the estimated age of the considered group of *Euplotes* spp. (86). Thus, our results indicate that the euplotid FSs are at an early stage of the FS accumulation process.

The number of euplotid FSs per genome at the equilibrium is expected to be between approximately 17 and 71 thousand. Thus, if we consider independent effects of FSs on fitness, the net lag load (loss of relative fitness) *L* conveyed by the total body of FSs becomes *L = 1* − (*1* − *S/N_e_*)*^F^*, which, depending on the *N_e_* value, may be either substantial and pose a potential hazard for the survival of *Euplotes*, or negligible. To assess this, we calculated the dependence between the net lag load of eventual frameshifts calculated from the obtained per-site 4*sN_e_* values and *N_e_*. We observe that the net lag load would drastically decrease fitness of *Euplotes* spp. on *N_e_* < 10^5^, whereas on *N_e_* > 10^6^ there is only a minor fitness decrease (*SI Appendix*, Fig. S6). Although the exact *N_e_* values of *Euplotes* spp. cannot be calculated in the absence of micronuclei genomic data, we may assume the euplotid *N_e_* values to be larger than 10^6^, as smaller organisms typically have much larger *N_e_* [for comparison, *N_e_* ~ 10^6^ for the current human population (87)]. Thus, although there will eventually be some significant load associated with frameshifts, it should not impact the survival of *Euplotes* spp.

## Discussion

The findings presented here suggest that *Euplotes* spp. are only starting their evolution towards a balanced use of frameshifting as a part of their genetic code. Since the ability of efficient frameshifting at internal stop codons is shared among all *Euplotes* spp., it likely existed in their last common ancestor. However, as we show here, the process of FS accumulation is very slow, and the number of currently observed FSs constitutes only about 4 to 20% of its expected maximum at equilibrium.

Is frameshifting itself useful for *Euplotes* spp.? Does it increase their fitness and at least partially compensate for the detrimental effects of FSs? It has been suggested that alternative genetic codes are frequently used in ciliates to protect their macronuclear genomes from foreign genetic elements (88). In addition, efficient frameshifting at out-of-frame stops in *Euplotes* spp. makes their genes resistant to single nucleotide insertions in protein-coding regions. Indeed, we found several instances of insertions that disrupt the protein-coding reading frame, but then the reading frame is restored due to frameshifting at a premature stop codon downstream (*SI Appendix*, Fig. S7). Thus, single nucleotide insertions in *Euplotes* protein-coding sequences result in a change of only a short segment of the encoded protein (between insertion and newly formed premature stop codon) rather than the entire downstream part of the protein.

In addition to these and other possible benefits of frameshifting, our study suggests an alternative, more likely evolutionary explanation of the abundant frameshifting in *Euplotes*. Once a species develops an ability to frameshift ribosomes at in-frame stop codons with high efficiency, the number of in-frame stop codons would start growing even if frameshifting is mildly deleterious. Once a certain number of FSs is reached, the process is expected to become entrenched and hence irreversible, as a reversal of the change that has enabled frameshifting would result in mistranslation of many genes (Fig. 2*F*). Hence, the high number of FSs in *Euplotes* does not necessarily imply that they are beneficial, but simply that they are not a limiting factor in the evolution and are not, individually or collectively, under sufficiently strong enough selection to be eliminated. However, depending on the long-term dynamics of strength and efficiency of selection, the exact frequencies of FSs occurrence in protein-coding sequences may vary. Thus we conclude that changes in the genetic code, even as profound as the violation of its triplet character, may be the result of neutral evolution. To what extent the standard genetic code is a product of adaptation and to what extent it is a product of neutral evolution possibly remains a matter of debate (89–93).

Our finding has an unexpected implication to the evolvability of the genetic code. Francis Crick’s Frozen Accident Hypothesis (94) was partly based on the necessity of the Discontinuity Principle. A change in the feature represented by a codon (amino acid or stop) would have sudden and severe consequences on the composition of the entire proteome to which a species would not be able to adapt in a short period of time (95). The later discoveries of many genetic code variants have revealed numerous possibilities for intermediate states such as ambiguous codons that prevent violations of the Discontinuity Principle (25, 27). Interestingly, the evolution towards frameshifting use as described here (Fig. 2*F*) does not violate the Discontinuity Principle since codons that specify (or alternatively “lead to”) termination of protein synthesis codons do not occur in protein-coding regions and the change of the meaning of UAG, UAA, and UGA at internal positions should not alter the composition of the proteome. It is the reversal process that would violate the discontinuity principle and thus should not be possible. A counterintuitive corollary of this asymmetry is that the number of organisms with such nontriplet features might increase with time.

## Materials and Methods

### Species Selection and Cell Cultures.

*Euplotes* strains used in this study were obtained from a large collection maintained in the laboratories of the Universities of Pisa and Camerino. Each species was selected based on the position it occupies within the *Euplotes* phylogenetic tree, which is commonly regarded as forming six major clades (numbered I to VI from the bottom) (*SI Appendix*, Fig. S3) (96, 97). *E. petzi* forms the most basal clade I. *E. raikovi* clusters with several other species into clade IV. *E. octocarinatus* and *E. harpa* lie in the well-supported clade V which includes most of the freshwater *Euplotes* species, but they belong to two different subclades. *E. euryhalinus*, *E. rariseta*, *E. minuta*, *E. crassus* and *E. focardii* cluster together into the poorly resolved and species-richest clade VI. However, these species branch into three different subclades, *E. euryhalinus* with *E. rariseta*; *E. focardii*; *E. minuta* with *E. crassus*. The latter two species are the most closely related among all the species analyzed here, as also demonstrated by previous breeding studies (98).

The selected *Euplotes* species have different ecologies. *E. octocarinatus* and *E. harpa* are temperate freshwater and brackish species, respectively (99, 100), while all the others are marine species. *E. focardii* is endemic to Antarctica (101). *E. petzi* and *E. euryhalinus* have a bipolar (Antarctic and Arctic) distribution (102, 103). *E. rariseta, E. crassus*, *E. minuta*, and *E. raikovi* are virtually ubiquitous in temperate coastal areas (104).

### RNA Preparation and Sequencing.

Cultures were grown under a daily cycle of 12 h of dark and 12 h of very weak light, at 4 to 6 °C (polar species) or 18 to 20 °C (nonpolar species) and fed on green algae *Dunaliella* (marine species) and *Chlorogonium* (freshwater species). They were expanded by daily food additions up to a cell density of about 10^4^ cells/mL, then washed free of food and debris, and re-suspended for 3 (temperate species) or 6 (polar species) d in fresh marine or distilled water before being harvested. The TRIzol plus purification kit (Thermo Fisher Scientific) was used to purify total RNA, following the manufacturer’s recommendations. Samples of about 10^7^ cells were concentrated by mild centrifugation and lysed by rapid resuspension in 1 mL TRIzol reagent containing phenol and guanidine. After chloroform addition and centrifugation, an equal volume of 70% ethanol was added to the aqueous phase containing RNA, which was next purified using silica cartridges. As a rule, an on-column DNase treatment was carried out for 45 min at room temperature to obtain DNA-free RNA preparations. After washing, RNA was eluted with 30 μL RNase-free water and stored at –80 °C before use. RNA concentration and purity were estimated by NanoDrop One Spectrophotometer (Thermo Fisher Scientific), while RNA integrity was analyzed by agarose gel electrophoresis.

cDNA library preparation and sequencing were carried out by BGI using Illumina TruSeq library construction and sequencing at Illumina HiSeq 2000 using 101PE.

### Assembly of Transcriptomes and Construction of Orthologous Gene Groups.

The resulting read libraries were trimmed with Trimmomatic (105) with parameters: ILLUMINACLIP:TruSeq3-SE:2:30:10 LEADING:3 TRAILING:3 SLIDINGWINDOW:4:15 MINLEN:36. The transcriptomes were assembled with Trinity (106) using the default set of parameters. For the expression analyses, the expression rate of each transcript was calculated as Transcripts Per Million (TPM) using the Kallisto software with the default parameters (107).

Orthologous gene groups (OGGs) were constructed using ProteinOrtho v. 5.15 (108) with parameters: −p = blastn −e = 1e−25 −identity = 70 on obtained transcriptomes.

The constructed OGGs were aligned with MUSCLE (109) with the default parameters. The coding regions were aligned by TranslatorX (110) with parameters: −p M −t F −w 1 −c 10. The predicted proteins were aligned with the Smith–Waterman algorithm (111).

To determine whether a sequence of a transcript is in the sense or antisense orientation, we relied on the locations of polyA and polyT tails, polyA tails at the 3′ ends were used as an indicator of the sense strand, while transcripts with polyT were classified as antisense and reverse complements of these transcripts were used instead. In the absence of polyA/T tails (truncated transcripts), we selected the orientation yielding the lowest number of indels in the longest ORF aligned to one of its orthologs.

### Calculation of Identity and d_N_/d_S_ Values.

To calculate pairwise protein identity, ORFs were translated using the euplotid genetic code (#10 from ncbi.nlm.nih.gov/Taxonomy/Utils/wprintgc.cgi) and aligned with the Smith-Waterman algorithm (111). All gap-containing positions were removed. The identity was calculated as the number of identical amino acids divided by the number of nongap positions in the alignment.

We employed two ways to calculate the *d*_N_*/d*_S_ ratio. The Nei-Gojobori method (112) yields values which can be straightforwardly compared by simple statistics (*SI Appendix*, *Supplementary Methods*). For the correction and validation of the obtained *d*_S_ values (Fig. 2*C*), we additionally calculated this statistic with the Nielsen–Yang method (113) implemented in the PAML software (114).

For the analysis of *d*_N_*/d*_S_ uniformity (*SI Appendix*, *Supplementary Methods*), random sampling was performed from compared ORFs (115). At each permutation round all four values needed to calculate the *d*_N_*/d*_S_ ratio, that is *dN*, *N*, *dS*, *S* [in the Nei−Gojobori notation (112)], were sampled from the respective Poisson distributions with parameters derived from the data. The *d*_N_*/d*_S_ ratios obtained for two ORFs were then compared. The percent of permutations which resulted in *d*_N_*/d*_S_ of adjacent (shorter) ORF being higher than *d*_N_*/d*_S_ of the primary (longer) ORF was used as the metric of uniformity.

### Transcriptome Quality Assessment.

Ciliates are obligate heterotrophs (116) mostly feeding on algae and other microorganisms, the remnants of which remain in their cytoplasm. They also may have intracellular symbionts (117). Hence the experimental separation of the ciliate DNA from the DNA of symbionts and prey is currently not feasible (118–120). This necessitated removal of contaminant sequences from assembled transcriptomes. We employed a four-step filtering procedure with subsequent controls to ensure that the transcripts considered in downstream analyses were indeed ciliate transcripts:

1. AT-content was calculated for each transcript and the resulting distribution was assessed (*SI Appendix*, Fig. S4). Since some distributions were bimodal, we filtered out all transcripts with the AT content less than the distribution mean minus 4 SD. This constraint follows observations that ciliate transcriptomes are AT-rich (121).
2. All transcripts having no homologs in other transcriptomes obtained in this study (singletons) were filtered out (see “*Assembly of transcriptomes and construction of orthologous gene groups*” above).
3. For each sequence, the nucleotide identity with its closest homolog from the sample was calculated. The resulting distribution appeared sharply trimodal (*SI Appendix*, Fig. S5) with ciliate sequences found only in the middle peak, as verified with BLASTn search (see below). From this distribution, we obtained the boundaries of the middle peak corresponding to the between-ciliate identity range. Then we discarded all transcripts with identity below the obtained lower bound of 0.65 and above the obtained upper bound of 0.95, which corresponded to the boundaries of the middle peak.
4. For the remaining sequences, the taxonomy of candidate contaminating species was identified. For this, we randomly selected 1,000 transcripts from each transcriptome and performed online BLASTn (122) search against the Genbank nonredundant database using the NCBI implementation at https://blast.ncbi.nlm.nih.gov/Blast.cgi. A nonciliate species was considered as being closely related to a contaminating species if it produced the best hit with at least 50% identity and alignment length of at least 50% relative to the query at E value below 10^−5^. A total of 41 contaminating (or closely related) organisms were identified (Dataset S5). All transcripts in our transcriptome were tested for sequence similarity to the genomic and transcriptomic sequences of these species using local BLASTn. All transcripts with hits exceeding 70% identity at E-value below 10^−25^ were removed.

To assess the reliability of this procedure, we searched the discarded sequences against known ciliate genomes and did not obtain significant alignments for any sequence from 111 discarded OGGs. We further queried sequences from the remaining 1,614 OGGs against the nonredundant Genbank database and did not obtain statistically significant alignments.

Finally, we checked for the presence of chimeric transcripts, i.e., transcripts assembled from reads originating from different organisms. We considered a transcript to be chimeric, if it yielded at least two BLAST hits (E-value < 10^−10^, Identity > 70%) that do not originate from the same organism with the overlap of at most 15 nt. No chimeric transcripts satisfying this criterion were identified.

Our contamination removal procedure and the restriction of the analyses to only genes with established orthologs produces a set of generally conserved genes with higher expression levels (*SI Appendix*, Fig. S9). Along with that, FSs tend to be more frequent in genes with lower expression levels (13), an effect that is also to some degree observed in our filtered transcriptomes (*SI Appendix*, Fig. S10). However, these effects should not result in substantial biases in our analyses, firstly, due to the weak FS tendency towards genes with low expression levels (*SI Appendix*, Fig. S10) and secondly due to FSs expected to have larger fitness effects in genes with higher levels of expression.

### Phylogenetic Analysis.

To validate the phylogenetic tree obtained using 18S rRNA (99) (*SI Appendix*, Fig. S3), we also constructed a tree using concatenated coding sequences obtained in this study. The tree was built using paralog-free OGGs containing genes from all nine studied euplotid species with the sequences from *Tetrahymena thermophila* as an outgroup. The coding regions were aligned with MUSCLE (109) and then the resulting alignments were concatenated. The tree was constructed with the Maximum Likelihood algorithm implemented in the PhyML package (123) using the automatic model selection feature (124) and 100 bootstrap replicates. To avoid biases in the estimation of neutral mutation rates arising from conserved regions in 18S rRNA, we estimated neutral divergence times from synonymous substitutions in protein sequences. Divergence times (per-branch *dS* values) were estimated with the m1 model of the PAML package. The ancestral sequences corresponding to frameshift sites were inferred using maximum parsimony (MP) (125, 126).

### Gains and Losses of Frameshift Sites.

To avoid misalignment errors we considered only frameshift sites within ±10 indel-free alignment blocks. The numbers of frameshift site gains and losses were calculated as the numbers of respective mutations, which all appeared as insertions and deletions of thymines in stop codons (Fig. 2*A*). Hence, the probability of frameshift site gain/loss at a specific context *i* was calculated as the number of gain/loss events *n* normalized over the number of the contexts *K*:

$$P_{i}=\frac{n_{i}}{K_{i}}$$

The contexts are defined by the codon preceding gained or lost stop codon, e.g., the context for frameshift sites is defined as NNN within NNN_TAR and in NNN_AR (potential gain upon insertion of T), R is purine (A or G), and the underscore separates codons in the coding reading phase.

### Calculating the Inflation of Frameshift Sites.

The temporal dynamics of the per-genome numbers of FSs is given by a logistic differential equation:

$$dF=\left(K-F\right)\;u_{\mathrm{gain}}\;dt\;-\;Fu_{\mathrm{loss}}\;dt\;=\;Ku_{\mathrm{gain}}\;dt\;-\;F\left(u_{\mathrm{gain}}+\;u_{\mathrm{loss}}\right)\;dt$$

where *F* = *F*(*t*) is the (variable) number of FSs, *u*_gain_ and *u*_loss_ are the rates of site gain and loss, respectively, and *K* is the (constant) number of suitable ancestral contexts for the shift gain. The solution is:

$$\begin{array}{c} F\left(t\right)\;=\left[F(0)\;-\;\frac{Ku_{gain}}{\left(u_{gain}\;+\;u_{loss}\right)}\right]\;\times \;exp[-(u_{gain}\;+\;u_{loss})t]\;+\frac{Ku_{gain}}{\left(u_{gain}\;+\;u_{loss}\right)}\;\; \end{array}$$

To simplify, let *A* = *Ku*_gain_*/(u*_gain_*+ u*_loss_), b = *Ku*_gain_ and F(0) be the current number of frameshift sites at *t* = *0*, then:

$$\begin{array}{c} F\left(t\right)\;=\;[F(0)-A]\;\times \;exp[-tb/A]\;+A\;\to \;A\;,\;as\;t\to \infty \; \end{array}$$

### Selection Inference.

We calculated the balance between selection and drift in the form of Kimura’s scaled selection coefficient *S* defined as *S* = *4sN*_e_, where *s* is the selection coefficient and *N*_e_ is the effective population size.

Next, we derive the expression to infer *S* from the ratio of FS gain and loss probabilities. The observed mutation rate under mutation, selection, and drift is (85):

$$\begin{array}{c} u\;=\;2N_{e}\mu [1\;-\;exp(-2s)]/[1-exp(-4N_{e}s)] \end{array}\;$$

where μ is the mutation rate.

The selection coefficient *s* can be presumed small, as events of frameshift site gain are observed. Thus, for *s*→0, 1 − exp(−2*s*) = 2*s*, and

$$\begin{array}{c} u\;=\;4N_{e}\mu s/\left[1-exp\left(-4N_{e}s\right)\right]\;=\;\mu S/\left[1-exp\left(-S\right)\right] \end{array}$$

As we are dealing with rare evolutionary events, i.e., indels and relatively small evolutionary times, the mutation rate may be presumed to equal the mutation probability: u = *P.*

Thus, the probability of FS gain is:

$$P_{gain}=P\left(S\right)=\frac{S{\text{μ}}_{gain}}{\left[1-\text{exp}\left(-S\right)\right]}$$

And, as the fitness effect of a frameshift site loss is equal to the negative fitness effect of a frameshift site gain, the probability of a frameshift site loss is:

$$\begin{array}{c} P_{\mathrm{loss}}\;=\;P\left(-S\right)\;=\;\left(-S\right)\mu_{\mathrm{loss}}/\left[1-exp\left(S\right)\right] \end{array}$$

The analysis of thymine indels in our data (*SI Appendix*, Fig. S8) suggests that μ_gain_ = μ_loss_. Thus

$$\begin{array}{c} P_{\mathrm{gain}}/\;P_{\mathrm{loss}}\;=\;-[1\;-\;exp(S)]/[1-exp(-S)] \end{array}$$

Or, alternatively:

$$S=ln\left(P_{\mathrm{gain}}/\;P_{\mathrm{loss}}\right)$$

To obtain the lag load of the total body of frameshift sites, we first calculated the expected total number of FSs at equilibrium by normalizing the numbers of genes in our samples containing FSs to the total number of genes estimated for *E. focardii* and *E. octocarinatus* (121) (*SI Appendix*, Fig. S6). Next, by presuming independent fitness effects of each frameshift, we calculated the net lag load of frameshift sites as the product of fitness effects of all sites.

### Equilibrium Frameshift Site Numbers.

The expected per-coding genome counts of FSs at mutational equilibrium were estimated from the calculated equilibrium numbers of FSs in our purified transcript sets and the total numbers of genes or the total coding bp numbers of *E. focardii* and *E. octocarinatus* estimated in ref. 121. The per-genome counts were obtained either as products of the equilibrium FS frequencies and coding genome lengths or as products of frequencies of genes containing FS at equilibrium and the total numbers of genes. The per-genome equilibrium counts were consistent between the approaches and between the two considered species (*SI Appendix*, Table S2).

## Supplementary Material

Appendix 01 (PDF)

Dataset S01 (CSV)

Dataset S02 (XLSX)

Dataset S03 (CSV)

Dataset S04 (GZ)

Dataset S05 (CSV)

## Data Availability

The RNAseq and RiboSeq data generated in the present study and the RiboSeq data generated in the previous study (13) have been submitted to SRA as bioproject PRJNA896607 (URL https://www.ncbi.nlm.nih.gov/bioproject/PRJNA896607). All scripts and data analysis protocols along with the constructed transcriptomes and tabulated frameshift sites are available online at https://github.com/sofyagdk/euplotes. All study data are included in the article and/or *SI Appendix*.
