# Supplementary material for: Nontriplet feature of genetic code in Euplotes ciliates is a result of neutral evolution
Source: Proc Natl Acad Sci U S A. 2023 May 22;120(22):e2221683120. doi: 10.1073/pnas.2221683120 (PMC10235951; doi:10.1073/pnas.2221683120)
Supplement: Supplementary file 1 — Appendix 01 (PDF) [file pnas.2221683120.sapp.pdf]

PNAS

Supporting Information for

Non-triplet feature of genetic code in *Euplotes* ciliates is a result of neutral evolution.

Sofya A. Gaydukova[1†,#], Mikhail A. Moldovan[2†,##], Adriana Vallesi[3], Stephen M. Heaphy[4,###], John F Atkins[4,5], Mikhail S. Gelfand[2] & Pavel V. Baranov[4*]*

[1] Faculty of Bioengineering and Bioinformatics, Lomonosov Moscow State University, Moscow, 199911, Russia

[2] A. A. Kharkevich Institute for Information Transmission Problems RAS, Moscow, 127051, Russia.

[3] Laboratory of Eukaryotic Microbiology and Animal Biology, School of Biosciences and Veterinary Medicine, University of Camerino, Camerino, 62032, Italy

[4] School of Biochemistry and Cell Biology, University College Cork, Cork, T12 XF62 Ireland.

[5] Department of Human Genetics, University of Utah, Salt Lake City, UT 84112.

*Corresponding authors: Mikhail S. Gelfand, Pavel V. Baranov

Emails: mikhail.gelfand@gmail.com, p.baranov@ucc.ie

[†]These authors contributed equally

[#] Present address: Institute for Medical Engineering and Science, Massachusetts Institute of Technology, Cambridge, MA 02139, USA

[##] Present address: Department of Biomedical Informatics, Harvard Medical School, Boston, MA 02115, USA

[###] Present address: National Clinical Trials Office, University College Cork, Cork, Ireland

This PDF file includes:

Supporting text
Figures S1 to S10
Tables S1 and S2
Legends for Datasets S1 to S5
SI References

Other supporting materials for this manuscript include the following:

Datasets S1 to S5

Supporting Information Text

Supplementary Methods

Pipeline for the efficient detection of frameshifting sites in *Euplotes* ciliates

Abbreviations and terms:

FS – frameshift site.

ORF – open reading frame, defined as a region between two in-frame stop codons.

TR – translated region (may contain FSs).

Longest ORF – longest ORF in a transcript with all six frames considered.

Primary ORF -- longest ORF in a stranded transcript in all three frames.

Adjacent ORF – ORF located next to a predicted TR.

Added ORF – adjacent ORF that satisfies the criteria and is added to the TR by the procedure.

Protein – putative protein, that is a formally translated amino acid sequence of a TR, taking into accounts FSs.

ORF product – amino acid sequence formally translated from a given ORF.

SD – standard deviation.

d_N/d_S – non-synonymous substitution rate divided by the synonymous substitution rate.

Pipeline parameters

1. Determinationing of the transcript strand

Transcript strands were determined as follows. If a transcript contained the poly-A tail either as a poly-A tail at the 3'-end or as a poly-T tail at the 5'-end, the transcript strand was determined accordingly. If no poly-A tail was present, we considered a pairwise alignment of the transcript with one of its orthologs in four combinations of orientations of two transcripts. In each set of four pairwise alignments, the longest conserved ORF was inferred and for this ORF the d_N/d_S values were calculated. Orientation corresponding to the lowest d_N/d_S value was then

selected. If the selected orientation was not consistent across orthologs, it was assigned by manual curation of the alignments.

2. General outline and the parameters

Stop codons in *Euplotes* spp, in addition to being signals of translation termination, can also function as ribosomal frameshift sites (1, 2) and may potentially be read through. To identify sequences of coding ORFs separated by possible frameshifting or stop-codon readthrough events, we rely on three features of ORFs known to be the hallmarks of coding sequences: (i) ORF length, (ii) protein identity between orthologs, (iii) signature of negative selection (low d_N/d_S). For each feature, we infer a set of thresholds based on the transcriptomic data (see below) and then demonstrate their validity and robustness by comparing the results to RiboSeq data and checking their adherence to known properties of euplotid frameshift sites. If an ORF does not satisfy any of the threshold-defined criteria, it is considered potentially non-coding. We consider only euplotid ORFs with established orthologous sequences in other *Euplotes* spp. and hence protein- and nucleotide sequence alignments may be constructed and, accordingly, parameters such as sequence identity or d_N/d_S may be obtained. We should note here that our method is expected to perform rather poorly on very short proteins (<30 amino acids), as identity and d_N/d_S values would not be reliably calculated.

The general outline of the algorithm is as follows. We start with the ORF in a transcript with the longest alignment with an ortholog and require it to be longer than a certain threshold. Next, we expand it on both sides, adding adjacent ORFs assuming +1 or +2 frameshifts or stop-codon readthrough, hence gradually constructing the translated region (TR). Hereinafter, for brevity, we refer to orthologous ORFs in alignments simply as ORFs. For each such expansion, we check that the added ORF is longer than another threshold. Both thresholds are selected so that the number of predicted frameshifts is not excessive. In addition, we check that the protein identity with orthologs is sufficiently high, and d_N/d_S is sufficiently low, using as a reference *Euplotes* genes without frameshifts. We also check that both values are relatively constant along the predicted TR, using parameters inferred by the analysis of a large set of mammalian proteins (for the identity), and the Poisson statistics for d_N/d_S. The details are given below.

The procedure used to set all thresholds aimed at robustness of the resulting set of FSs. Robustness of the results was defined as the absence of substantial changes in the set of predicted FSs upon minor changes of the thresholds. The thresholds were optimized sequentially: first, we looked into stability of the number of predicted FSs with respect solely to the length thresholds (Suppl. Fig. S1c). Next, having selected length thresholds yielding stable results, we applied them with different combinations of thresholds on the uniformity of the protein identity and d_N/d_S . The final set of thresholds (listed in Table S1) was used to predict FSs on which the main results were obtained.

3. Parameters selected using the transcriptomic data

3.1. Primary ORF and its extension

In each transcript with the determined strand we selected an ORF such that (i) its length exceeded a given threshold (30 amino acids) and (ii) after formal translation it provided the longest amino acid alignment with a transcript from a different *Euplotes* species. The threshold for the length of primary ORFs was established by the same procedure as the one used to establish other threshold lengths (see below). These *primary ORFs* turned out to be long, demonstrating high sequence identity and low d_N/d_S (Suppl. Fig. S1f-i), hence we considered them to be *bona fide* protein-coding ORFs.

The TR is then constructed by extending the primary ORF with shorter ORFs adjacent at the 5'- and 3'-ends. All three reading frames are considered, hence accounting for stop codon

readthrough and +1 and +2 frameshifting. If adjacent ORFs in several reading frames satisfied the length, identity, and d_N/d_S conditions (see below), we used the longest ORF if its length was more than 10/9 of the second-longest ORF, otherwise we selected the added ORF based on higher protein identity to the closest ortholog.

3.2 Extending ORFs – length thresholds

The number of predicted FSs depends on the minimal-length thresholds for adjacent ORFs (Suppl. Fig. S1c). This dependency is particularly strong when the length threshold is low. This is caused by a combinatorial explosion in non-coding ORFs erroneously added to TRs. On the other hand, while high thresholds yield high-confidence FS predictions, many genuine FS are not detected. We selected thresholds, 25 and 15 codons for the downstream and upstream TR extension, respectively, which corresponded to the sharpest changes in the curve slopes, so as to yield the maximal number of FSs with the minimal number of false negatives.

Additionally, we set a soft threshold for the minimal length of adjacent ORFs based on the distributions of protein identity values of the adjacent ORFs (Suppl. Fig. S1de). That is, we allowed a longer adjacent ORF to be added to the TR if it passed the conditions on the identity level. The rationale for that is that, while at the N- and C-ends of the protein alignments the identity levels tended to deviate largely for small window sizes, for larger windows (approximately >60 amino acids), this effect became negligible (Compare Suppl. Fig. S1g and Suppl. Fig. S1de). Thus, the longer ORFs were subjected solely to the threshold-based identity checks, and the shorter, to the checks of the uniformity of identity (see below).

3.3 Protein identity thresholds

For long adjacent ORFs (with lengths above the soft threshold), we apply the threshold equal to the lowest observed protein identity for primary ORFs, that is 0.65. In this case it is impossible to apply the filter on uniformity of identity along the protein sequence, as only few non-overlapping windows of the size equal to the ORF length fit into the protein, and the distribution of identity may not be built on these few data points.

Conversely, for short adjacent ORFs (with lengths between the hard and soft thresholds), the threshold on identity cannot be set, as identities at N- and C-termini may be lower than those of the middle parts of a protein (Suppl. Fig. S1de). However, a relaxed identity-based criterion can be set here, based on the prediction of identity at the termini given identity in the middle. For that, we considered 20317 (3) well-annotated groups of mammalian orthologs from the OMA database and aligned them with ClustalΩ (4). For each group of orthologs, we constructed a distribution of identities calculated in non-overlapping windows of size n (ranging from 3 to 60 amino acids), situated between nucleotides n and $L–n$, L being the alignment length, that is, in the middle part of the alignment. For each such distribution we used the obtained mean μ and standard deviation σ to obtain Z-scores for identity calculated in the remaining N- and C-terminal windows. Next, for each window size n, we constructed distributions of the obtained Z-scores across groups of orthologs and calculated one-sided 95% confidence intervals for these distributions (Suppl. Fig. S1ab). In the alignments of euplotid transcripts, if the observed Z-score of the difference in the identity in an adjacent ORF and the predicted translated region was in the constructed 95% confidence interval for the respective window size, this ORF was accepted as an extension of the TR, and the respective FS or codon readthrough event was predicted. Otherwise this ORF was filtered out as non-coding.

3.4 d_N/d_S uniformity threshold

Our d_N/d_S uniformity threshold was defined as follows. The d_N/d_S values were calculated separately for the longest ORF and for an added ORF. Next, the obtained rates were compared with a permutation statistic and the respective p-value was obtained (Suppl. Fig. S1i). This

p-value was considered as the uniformity parameter, for which a threshold value 0.85 was obtained from the bimodal distribution produced by all adjacent ORFs (Suppl. Fig. S1i). At that, as the d_N/d_S rates of the primary ORF and the extending ORFs are expected to come from the same distribution, only the left peak of the *p*-value distribution is considered.

4. Pipeline

The resulting procedure is as follows:

1. For each stranded transcript from each purified euplotid transcriptome (see Methods: Transcriptome quality assessment), the euplotid ortholog from our dataset yielding the longest nucleotide alignment is obtained. If no ortholog has been found, the transcript is discarded. All nucleotides with no alignment to an orthologous sequence are discarded. If the primary ORF of the sequence is not aligned with the primary ORF of an ortholog, the transcript is discarded.

2. For the primary ORF, a codon-wise alignment is built with TranslatorX(5). The d_N/d_S rate of the longest ORF is calculated (see Materials and Methods: Calculation of identity and d_N/d_S values). At this stage, we consider the primary ORF as a translated region (TR) and initialize the extension procedure, an iteration of which is described below (steps 3-5).

3. ORFs corresponding to +1 and +2 frameshifting and stop-codon readthrough at the ends of the TR are considered. For each such adjacent ORF, its length is calculated. If the length of an adjacent ORF is smaller than the hard threshold of 15 amino acids for the 5'-end ORFs and 25 amino acids for the 3'-end ORFs, the ORF is discarded.

4. For each remaining adjacent ORF, a codon-wise alignment with the ortholog is built and the protein identity and d_N/d_S rates are calculated.

5. If the length of an adjacent ORF exceeds the soft length threshold of 60 codons, the minimal protein identity threshold of 0.65 is applied to the product of its translation. If this criterion is not satisfied, the ORF is discarded.

6. If the length of an adjacent ORF is below the soft length threshold, the *Z*-score is calculated for the adjacent ORF protein identity value based on the parameters of the identity distributions for the entire TR. The *Z*-score threshold is determined from the length of an adjacent ORF using our results obtained for mammalian orthologs. ORFs with the *Z*-scores higher than obtained thresholds are discarded.

7. If the length of an adjacent ORF is below the soft length threshold, the d_N/d_S uniformity parameter is calculated. If the calculated value is below 0.85, the respective ORF is discarded.

8. If by this step no adjacent ORFs have passed filters 3, 5, 6, and 7, the TR is returned for the regarded transcript. Otherwise, among remaining adjacent ORFs, the longest one is selected and assumed translated with the respective event (+1 or +2 frameshifting or stop-codon readthrough). The ORF is added to the TR and steps 3-8 are repeated.

9. The resulting set of translated ORFs constituting the TR and respective FS or stop codon readthrough events are the output.

Parameters used to predict TRs are summarized in Supplementary Table S1.

Validation

5. Validation based on *E. crassus* RiboSeq data

To validate the procedure for predicting coding sequences and the obtained set of parameters, we used a RiboSeq dataset of *E. crassus* (see Materials and Methods: Sample collection and library preparation). Predicted coding ORFs for *E. crassus* can be verified by mapping the RiboSeq reads on the transcript. For each putatively coding ORF, if it is covered with a sufficiently high number of RiboSeq reads, the prediction is considered as true positive. Conversely, if an ORF in a predicted TR is not covered with reads or is covered with a significantly lower density of reads than the primary ORF, the prediction is considered a false positive. If a high number of reads covers a predicted untranslated region, it is considered a false negative. Otherwise, the prediction is true negative. Unfortunately the triplet periodicity of the RiboSeq data (13) was insufficient for the inference of the translated reading frame. Therefore we estimated only the ability of the algorithm to predict coding sequences regardless of the frame.

We analyzed how each parameter (Suppl. Tab. S1) influences the true-positive rates (TPRs) and the false-positive rates (FPRs) of +1 FS events in *E. crassus*, while keeping other parameters constant (Suppl. Fig. S2). Our parameters, selected independently using transcriptomic data, yield the cumulative TPR of 0.543 for 5'-end ORFs and of 0.508 for 3'-end ORFs, and the net FPR of 0.0.

6. Validation based on known properties of euplotid FSs

In previous studies, euplotid FSs were predicted in 2–30% of transcripts and shown to involve a specific A-rich trinucleotide motif (1, 6, 7). Using our algorithm, we predicted frameshift sites in 3.9% of transcripts (192 sequences out of 4903). At that, stop codons identified as *Euplotes* FSs feature the same strong nucleotide context as the one reported previously (1) (Figure 1b), whereas stop-codons predicted to be signals of translation termination do not fall within this motif (Figure 1b), as expected.

7. Validation based on known properties of euplotid transcripts

The d_N/d_S values of the predicted TRs fell into the 95% confidence interval of [0.044, 0.324] with the mean of 0.163, being similar to the ones calculated for model organisms, e.g. for the human population (8).

In addition, while the AT-content differed in coding regions, 3'-UTRs, and 5'-UTRs, there were no differences in the AT-content between the regions upstream and downstream of FSs (Figure 1e). This result fell in line with previously reported patterns of nucleotide composition around start and stop codons in ciliates (1, 7). The predicted 3'-UTRs, i.e. the sequences between the terminal stop codon and the poly-A tail, were extremely short, being on average 33 nt, and featured an enhanced density of stop codons, as expected (9) (Figure 1gh).

Code availability

The pipeline implementation written in Python 3.7 along with the data analysis protocols are available at https://github.com/sofyagdk/euplotes.

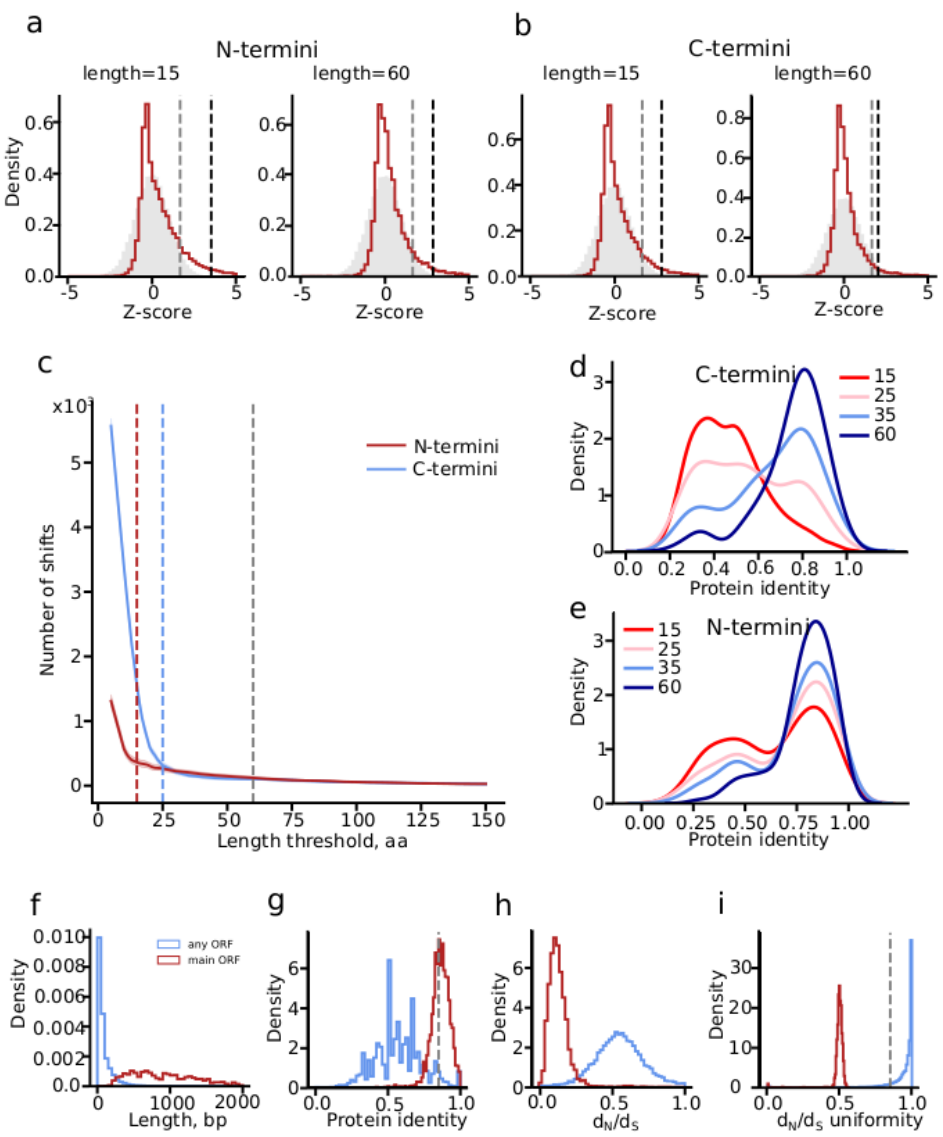

Fig. S1. Algorithm parameters. (ab) Distributions of *Z*-scores calculated for terminal protein regions based on the parameters of the distributions of identities calculated for internal regions of mammalian protein alignments (see the text for details). Grey histogram is the normal distribution, note the heavier right tail of obtained distributions. Dotted lines show 95% quantiles for the normal (light gray) and for the obtained (dark gray) distributions. **(a)** Distributions for N-ends **(b)** Distributions for C-ends. **(c)** Number of predicted frameshifts as a function of the length threshold. Colored dotted lines show hard thresholds for N- and C-ends. Grey dotted line shows the soft threshold (see text) **(de)** Protein identity distribution of added ORFs at different length thresholds **(f)** Distributions of lengths for main ORFs (red) and randomly selected ORFs (blue). **(g)** Protein identity distributions for main ORFs (red) and randomly selected ORFs (blue). **(h)** d_N/d_S distributions for main ORFs (red) and randomly selected ORFs (blue). **(i)** The number of ORFs with different d_N/d_S uniformity parameters for adjacent ORFs (red) and primary ORFs (blue). The selected threshold of 0.85 is indicated with the dashed line.

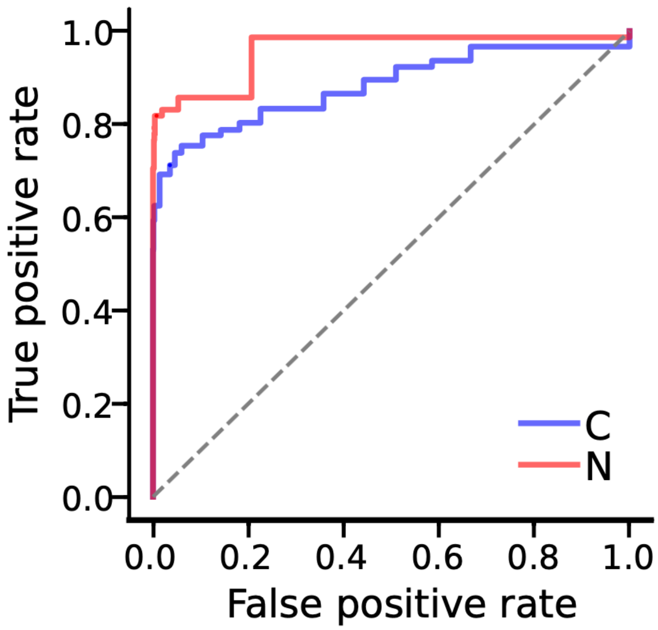

Fig. S2. Receiver operating characteristic (ROC) curves for the ORF length parameter used in TR prediction for C- and N-end extension

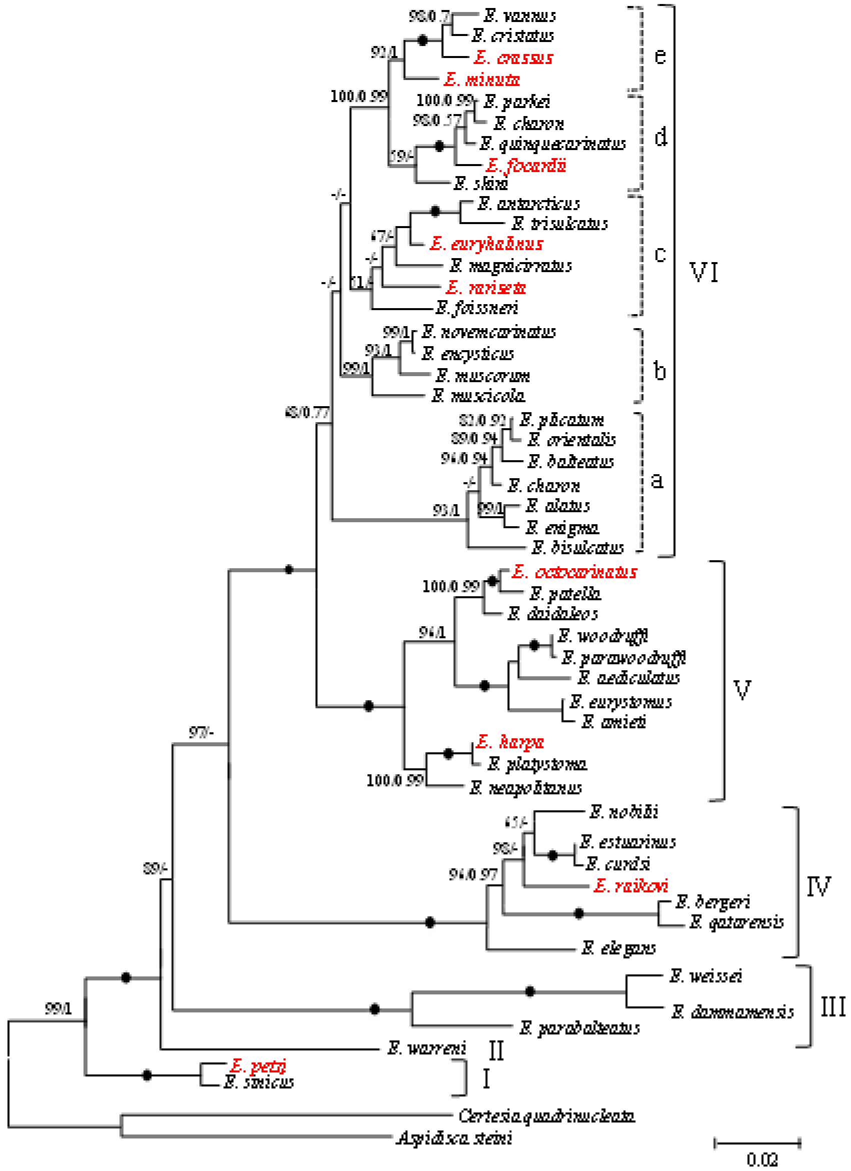

Fig. S3. Phylogenetic tree based on 18S rRNA of *Euplotes* spp. The *Euplotes* species from this study are shown in red. Numbers near the branches represent bootstrap values of the Maximum Likelihood and Posterior Probability of Bayesian inference analyses, respectively. Fully supported (100/1.00) branches are marked with solid circles. All branches are drawn to scale. The scale bar corresponds to two substitutions per 100 nucleotide positions. Modified from Valbonesi et al., 2021 (10).

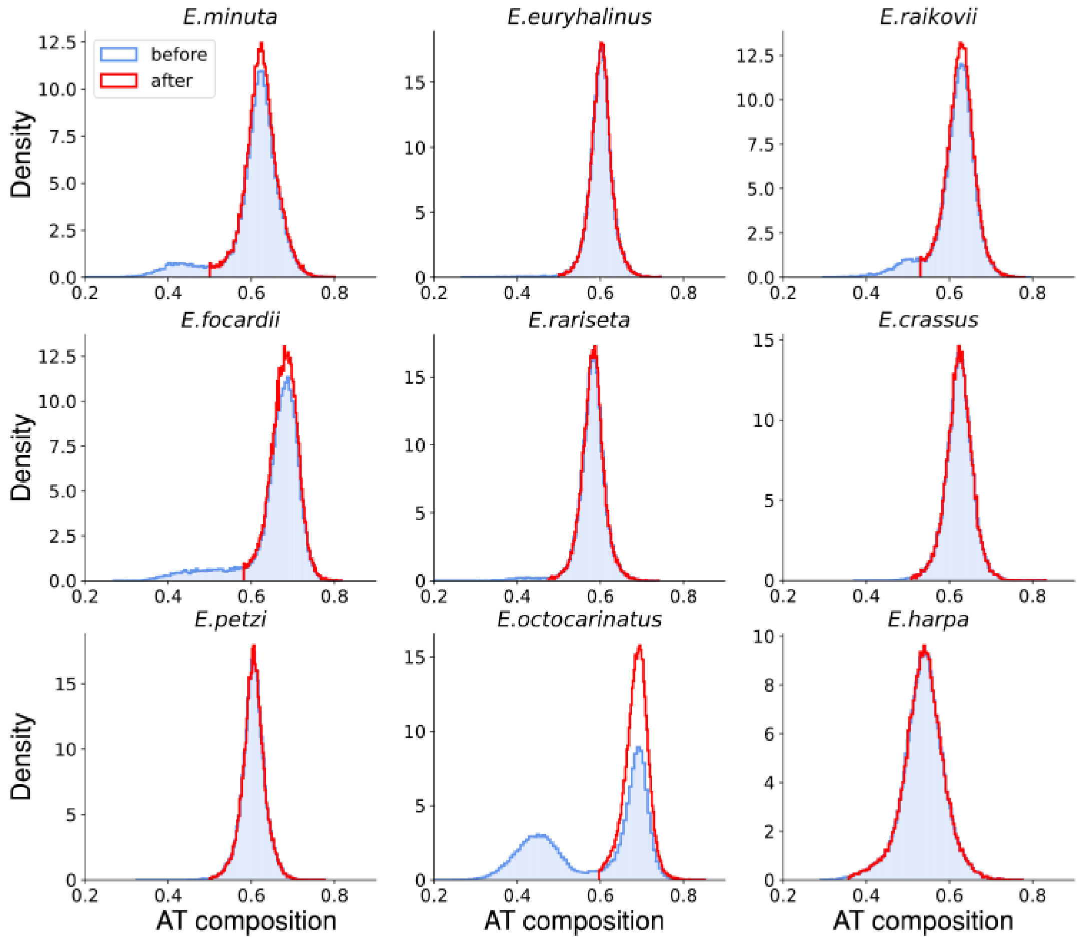

Fig. S4. Transcriptome quality assessment based on AT content. Distributions of AT-compositions calculated in each transcript for each *Euplotes* species before (blue) and after filtering (red), see Methods.

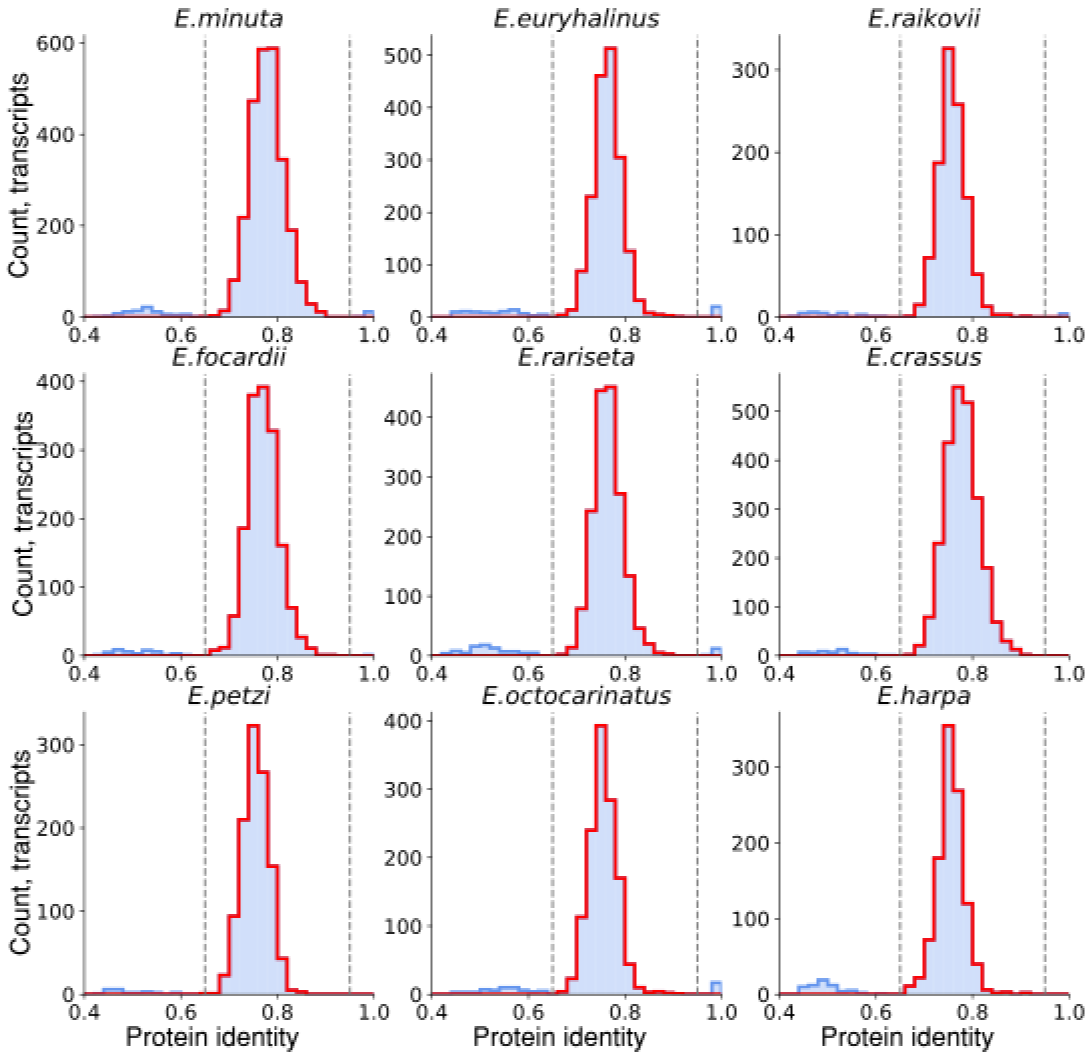

Fig. S5. Transcriptome quality assessment based on sequence similarity with orthologs. Distributions of pairwise nucleotide identity for transcripts before (blue) and after (red) filtering for each *Euplotes* species.

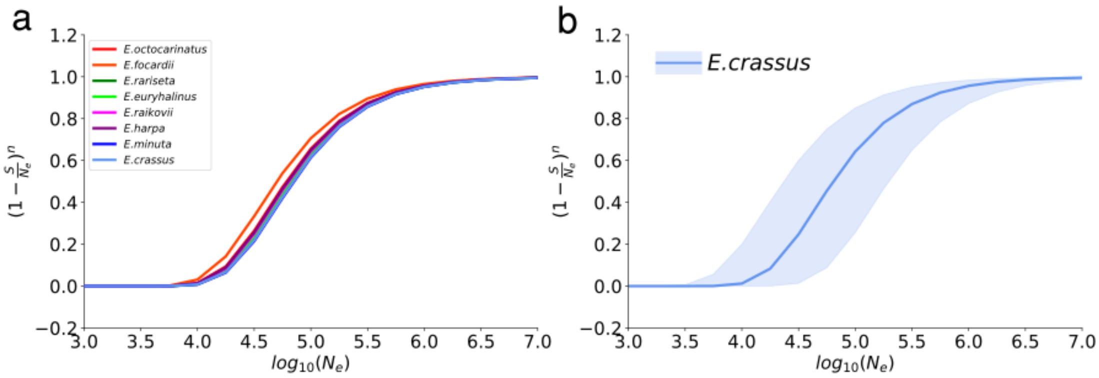

Fig. S6. Selection against the total body of euplotid frameshift sites when the equilibrium number of frameshifts is reached. (a) Dependency between the effective population size and normalized lag loads induced by frameshifts in all considered *Euplotes* species **(b)** Confidence interval for the dependency shown in panel (a) for *E. crassus*.

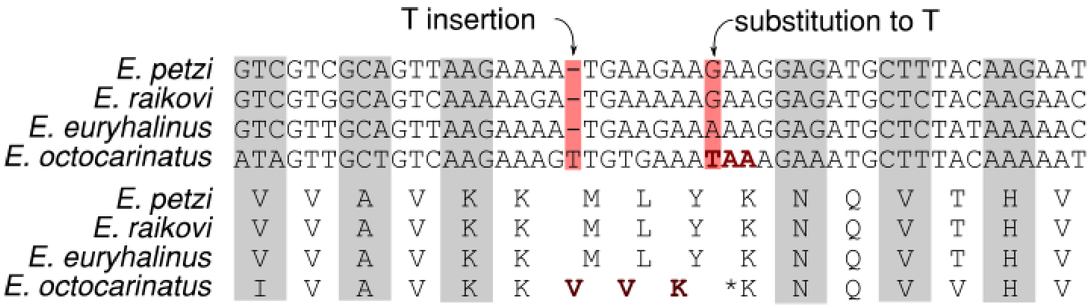

Fig. S7. An alignment fragment showing restoration of the initial reading frame through ribosomal frameshift. (top) Single-nucleotide insertion in *E. octocarinatus* and a +1 frameshift site 8 bp downstream restoring the initial reading frame **(bottom)** Respective protein sequence alignment. Note the substitution of MLY tripeptide with VVK.

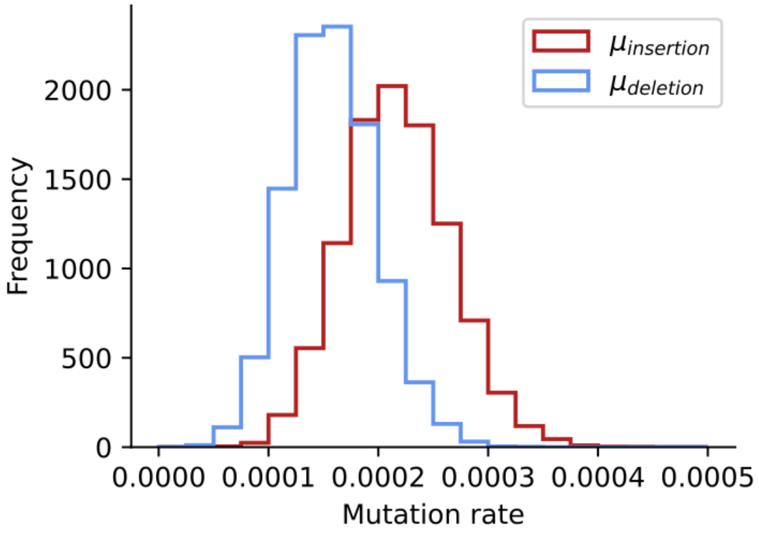

Fig. S8. Thymine insertion and deletion rates. Histograms show bootstrap by orthologous gene alignment sets of per-nucleotide insertion (red) and deletion (blue) rates of thymine in non-coding regions. Permutation test P-value showing the absence of significant differences in mutation rates is also shown.

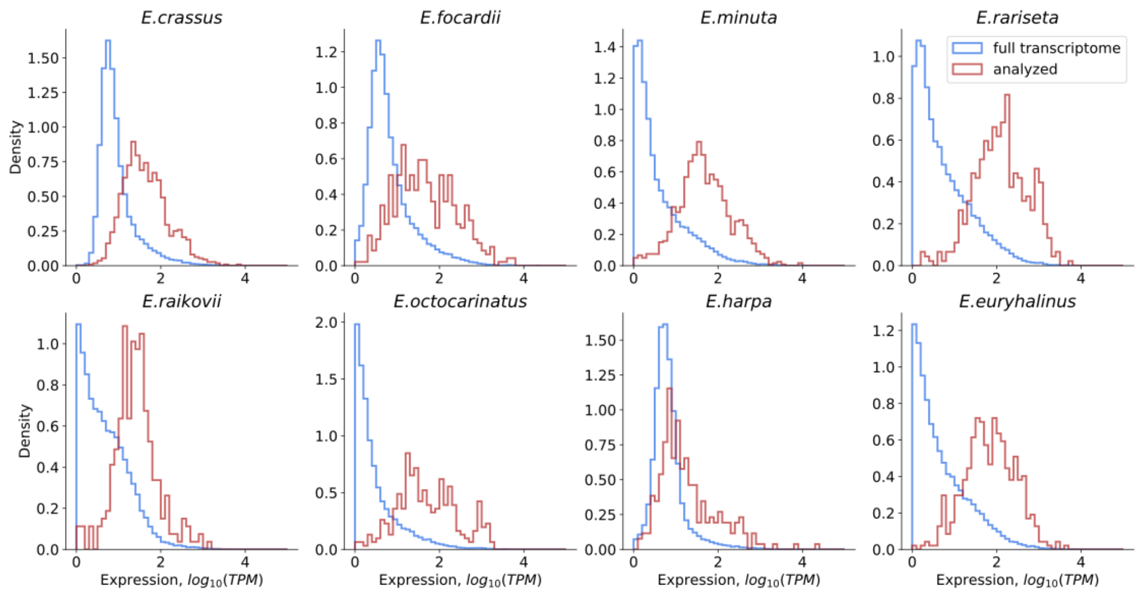

Fig. S9. Differences in expression rate distributions in analyzed gene set (red) vs. transcriptome-wide distributions of expression rates (blue).

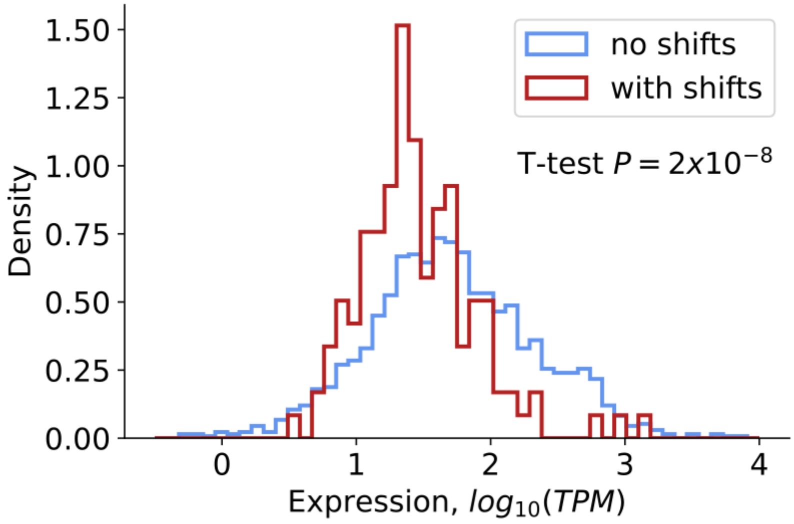

Fig. S10. Expression rates of transcripts containing (red) and not containing frameshifts (blue). The P-value of t-test demonstrating significantly lower expression rates in genes with frameshifts is also shown. This result is consistent with the results reported in Suppl. Ref. (1).

Table S1. All parameters used in the pipeline for prediction of TRs.

**Parameter**	**Value**
Longest length threshold	30 codons
Longest ORF identity	0.64
Threshold for the uniformity of protein identity	Defined as shown in Suppl. Fig. S1i
Soft length threshold	60 codons
Hard length threshold	15 codons (for the 5’-end), 25 codons (for the 3’-end)
d_N/d_S uniformity threshold	0.85

Table S2. Prediction of the frameshift site counts at equilibrium. Gene counts per genome and gene lengths were obtained from Ref. 119.

	E.octacarinatus (2.29 - 9.69 fold increase in FS sites)		***E.focardii*** (3.82 - 17.02 fold increase in FS sites)	
	Per-genome prediction	**# in analyzed data**	***Per-genome prediction***	**# in analyzed data**
Gene number	29076	317	28881	473
Average gene length (bp)	1178	1145.48	1085	620,4
Per-genome coding length (bp)	34251528	363117	31335885	293472
Current # of FS sites inferred from gene counts	2476.5	27	1221.2	20
Current # of FS sites inferred from coding bp counts	2546.84	27	2135,5	20
FS sites #, 5th percentile inferred from gene counts	5659.7	61.7	4665.0	76.4
FS sites #, 95th percentile inferred from gene counts	24004.0	261.7	20784.8	340.4
FS sites #, 5th percentile inferred from coding bp counts	5820.4	61.7	8157.7	76.4
FS sites #, 95th percentile inferred from coding bp counts	24685.5	261.7	36731	340.4

Dataset S1 (separate file). Coding regions of 4903 sequences studied

Table containing 6 columns. Each row corresponds to a sequence from the orthology group alignments. column “orthology_filename” contains the unique filename of the orthology group file (Dataset S4), column “orthology_filename” contains a unique identifier of the sequence (same as in Dataset S5-S6), column “coding_region” contains hyphen-separated boundaries of transcribed regions, the even positions corresponding the start of the region (0-based), the odd positions corresponding to the position of stop codons. The column “main_frame” contains the hyphen-separated boundaries of the primary coding frame. The other two columns are the same as “coding_region” and “main_frame”, but the coordinates of the coding regions are given for the respective columns in the alignment files.

Dataset S2 (separate file). Coding regions of Sequences with frameshifts

Table contains sequences with frameshift sites. The first five columns are the same as in Dataset S1, the last column shows the number of frameshift sites in the particular sequence.

Dataset S3 (separate file). Orthologous sequence identifiers

Table containing 11 columns, rows correspond to orthology groups. Column “filename” contains the filename containing the alignment of the orthologous group, column “len” contains a number from 2 to 9 which shows the number of sequences in the orthology group. The remaining nine columns contain a unique sequence identifier of the sequence of the particular species, or None if the orthology group does not contain a sequence from this species.

Dataset S4 (separate file). Alignments of orthologous sequences

Archive named ortholog_alignments.tar.gz contains a folder with files. Each file contains one ortholog gene group, each ortholog gene group consists of 2 to 9 orthologous sequences of different *Euplotes* species analyzed.

Dataset S5 (separate file). Contamination information. Table with contaminating species, the accession codes of tested sequences, and the number of euplotid transcriptome sequences similar to some sequence in the contamination dataset, given for each species separately.

SI References

1. A. V. Lobanov, *et al.*, Position dependent termination and widespread obligatory frameshifting in *Euplotes* translation. *Nat. Struct. Mol. Biol.* **24**, 61–68 (2017).
2. J. F. Atkins, G. Loughran, P. R. Bhatt, A. E. Firth, P. V. Baranov, Ribosomal frameshifting and transcriptional slippage: From genetic steganography and cryptography to adventitious use. *Nucleic Acids Res.* **44**, 7007–7078 (2016).
3. A. M. Altenhoff, *et al.*, The OMA orthology database in 2015: function predictions, better plant support, synteny view and other improvements. *Nucleic Acids Res.* **43**, D240–D249 (2015).
4. F. Sievers, *et al.*, Fast, scalable generation of high-quality protein multiple sequence alignments using Clustal Omega. *Mol. Syst. Biol.* **7**, 539 (2011).
5. F. Abascal, R. Zardoya, M. J. Telford, TranslatorX: multiple alignment of nucleotide sequences guided by amino acid translations. *Nucleic Acids Res.* **38**, W7-13 (2010).
6. L. A. Klobutcher, Sequencing of Random *Euplotes crassus* Macronuclear Genes Supports a High Frequency of +1 Translational Frameshifting. *Eukaryot. Cell* **4**, 2098–2105 (2005).
7. R. Wang, J. Xiong, W. Wang, W. Miao, A. Liang, High frequency of +1 programmed ribosomal frameshifting in *Euplotes octocarinatus. Sci. Rep.* **6**, 21139 (2016).
8. P. C. Ng, *et al.*, Genetic Variation in an Individual Human Exome. *PLOS Genet.* **4**, e1000160 (2008).
9. E. C. Swart, V. Serra, G. Petroni, M. Nowacki, Genetic Codes with No Dedicated Stop Codon: Context-Dependent Translation Termination. *Cell* **166**, 691–702 (2016).
10. A. Valbonesi, G. Di Giuseppe, A. Vallesi, P. 2020 Luporini, Two new species of Euplotes with cirrotype-9, *Euplotes foissneri sp. nov.* and *Euplotes warreni sp. nov.* (*Ciliophora, Spirotrichea, Euplotida*), from the coasts of Patagonia: implications from their distant, early and late branching in the *Euplotes* phylogenetic tree. *Int. J. Syst. Evol. Microbiol.* **71**, 004568.
11. M. Mozzicafreddo, *et al.*, The macronuclear genome of the Antarctic psychrophilic marine ciliate *Euplotes focardii* reveals new insights on molecular cold adaptation. *Sci. Rep.* **11**, 18782 (2021).
